# This girl is on fire

**DOI:** 10.1192/j.eurpsy.2021.660

**Published:** 2021-08-13

**Authors:** P. García Vázquez

**Affiliations:** Psiquiatría, Complejo Asistencial Universitario León, León, Spain

**Keywords:** Burning mouth syndrome, Quetiapine

## Abstract

**Introduction:**

Burning mouth syndrome (BMS) is a chronic condition characterized by a burning sensation of the oral cavity and is often associated with taste disturbances and xerostomia.

**Objectives:**

To study the psychotropics as part of the possible etiologies of this síndrome.

**Methods:**

A 67-year-old woman complained of burning pain in the tongue and oral mucosa, taste disorder, and sensory impairment. Slight improvement after treatment with Gabapentine 300mg (1-0-0). The pain is constant, with sharp characteristics. Improves when eating, the ability to taste is preserved. Subsequently, treatment with 2% lidocaine rinse (3-4 times / day) is tested, with temporary pain relief. Lorazepam 1mg (1-0-0), without improvement. Patient in follow-up by the Neuropsychiatry consultation for 3 years, due to major depressive disorder in treatment with Quetiapine 100mg (0-0-1).

**Results:**

In the first consultation the treatment is modified, adding Duloxetine 60 mg (1-0-0) and Alprazolam 0.5mg (1 / 2-1 / 2.0), and reducing the dose of Quetiapine to 75 mg and then 50mg. In the subsequent consultation, one month later, she only manages to reduce the neuroleptic dose by half, without noticing clinical improvement. After two months, she has completely removed the quetiapine, and completely disappearing the burning mouth sensation, improving his affective clinic in the same way.

**Conclusions:**

There are a large number of drugs that produce xerostomia, in intimate relationship with the burning mouth. Among those we can find antihistamines, neuroleptics, antihypertensives, and benzodiazepines. In many cases, correcting or eliminating these etiologic agents does not improve or stop the initial symptoms, but sometimes, it does.

